# Virtual Axle Detector Based on Analysis of Bridge Acceleration Measurements by Fully Convolutional Network

**DOI:** 10.3390/s22228963

**Published:** 2022-11-19

**Authors:** Steven Robert Lorenzen, Henrik Riedel, Maximilian Michael Rupp, Leon Schmeiser, Hagen Berthold, Andrei Firus, Jens Schneider

**Affiliations:** 1 Institute for Structural Mechanics and Design, Technical University of Darmstadt, 64287 Darmstadt, Germany; 2 iSEA Tec GmbH, 88046 Friedrichshafen, Germany

**Keywords:** moving load localisation, nothing-on-road, free-of-axle-detector, bridge weigh-in-motion, structural health monitoring, field validation, continuous wavelet transformation, machine learning, fully convolutional networks

## Abstract

In the practical application of the Bridge Weigh-In-Motion (BWIM) methods, the position of the wheels or axles during the passage of a vehicle is a prerequisite in most cases. To avoid the use of conventional axle detectors and bridge type-specific methods, we propose a novel method for axle detection using accelerometers placed arbitrarily on a bridge. In order to develop a model that is as simple and comprehensible as possible, the axle detection task is implemented as a binary classification problem instead of a regression problem. The model is implemented as a Fully Convolutional Network to process signals in the form of Continuous Wavelet Transforms. This allows passages of any length to be processed in a single step with maximum efficiency while utilising multiple scales in a single evaluation. This allows our method to use acceleration signals from any location on the bridge structure and act as Virtual Axle Detectors (VADs) without being limited to specific structural types of bridges. To test the proposed method, we analysed 3787 train passages recorded on a steel trough railway bridge of a long-distance traffic line. Results of the measurement data show that our model detects 95% of the axles, which means that 128,599 out of 134,800 previously unseen axles were correctly detected. In total, 90% of the axles were detected with a maximum spatial error of 20 cm, at a maximum velocity of $v_{\max}=56.3\;m/s$. The analysis shows that our developed model can use accelerometers as VADs even under real operating conditions.

## 1. Introduction

All over the world, aging bridge infrastructure is facing the challenge of increasing traffic loads. For example, in the United States, there are more than 617,000 bridges, of which 42% are at least 50 years old and 7.5% are considered structurally deficient [1]. In Germany, more than 40% of the 25,710 railway bridges are older than 80 years, while the average lifespan is about 122 years [2,3]. The application of structural health monitoring (SHM) makes it possible to increase the operational availability and safety of these structures. Since knowledge of the actual operational loads is of high importance for the condition assessment of the structures, especially when it comes down to the assessment of fatigue failure and the evaluation of the remaining service life, the determination of the loads is a key aspect in the field of SHM. Since the direct measurement of loads is often technically difficult and usually requires significant financial resources [4,5,6], different methods for load identification based on measured structural responses have been developed [6,7,8,9,10]. In the case of bridges, these methods are referred to as Bridge Weigh-In-Motion (BWIM) [11,12,13,14].

For the majority of BWIM systems, information about the vehicle configuration (number of axles and axle spacing) and velocity is a prerequisite [14]. For this purpose, conventional axle detectors are used [5,15,16,17]. However, due to the impact loads of the wheels, axle detectors have a limited durability [18]. In addition, the installation of the axle detectors always implies road or railway track closures. The latter case especially requires a considerable amount of bureaucratic, logistic, and financial effort. To avoid these issues, modern BWIM systems use axle detection concepts that use only sensors installed under the bridge. These concepts are called nothing-on-road (NOR) or free-of-axle-detector (FAD) [19,20].

FAD technology uses two additional strain sensors at different positions on the bridge to determine vehicle configuration and speed [20]. Since FAD is only suitable for specific types of bridges [14], it was investigated whether the axle velocity and the axle spacing could be determined using global flexural strain or shear strain measurements [14,21]. For the proposed method in [21], which makes use of shear strains, the application of strain gauges at the level of the neutral axis is required. This is a challenge for complex structures, especially for railway bridges with ballasted tracks, because in such cases, the position of the neutral axis cannot be easily determined. However, the proposed method in [14] is only suitable for structures where the structural response is dominated by the quasi static response of the bridge, e.g., where the dynamic amplification is low. In addition, the second time derivative of the strain signals is used here; this makes the method sensitive to measurement noise, which entails the need for a suitable noise filter, depending on the application.

In [14], the method of virtual axles was proposed. It assumes a vehicle with many virtual axes, where all axles, except for the real ones, are weightless. The true axles and their weights are then determined by solving a constrained least square problem. As the authors stated, the method fails when there is significant noise in the signals. Since a significant amount of noise is present in field measurements and other practical applications, the method cannot be practically applied without a sophisticated regularisation method. Furthermore, the method uses experimentally determined lines of influence, so it is not applicable to cases with significant dynamic amplification in their structural response.

To the best of our knowledge, only Zhu et al. [22] have published a study on the accelerometer-based axle detection method so far. Here, a shallow Convolutional Neural Network (CNN) is used to detect potential axle sequences, which are then transformed with a continuous wavelet transform. Afterwards, the axles are detected in the transformed signals with the use of peak-finding methods. The method of Zhu et al. [22] requires accelerometers close to the supports. The acceleration signals of these sensors are dominated by the vehicle-induced impulses when entering and leaving the bridge, leading to the clear axle recognition in the time domain. In contrast to this method, our approach allows for the use of sensors at arbitrary positions, effectively reducing the number of sensors required by SHM applications.

An axle detection method based on acceleration measurements is desirable because the installation of acceleration sensors is much easier and less laborious compared to strain gauges. However, accelerometers are often already installed on the structure to determine the modal parameters and are not necessarily located close to the supports. Therefore, we propose a method that would enable accelerometers arbitrarily placed on a bridge to be used as VADs. In this way, the same acceleration sensors used for analysing the global structural behaviour (e.g., at midspan or quarter span of beam-like bridges) can also be employed in axle detection without having to install additional sensors in the proximity of the supports.

**Figure 1:**
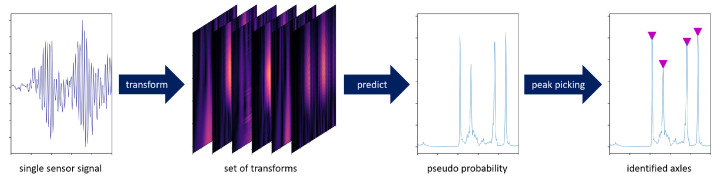
VAD process, from left to right: acceleration signals from a single sensor, set from different transformations of the signal, localization estimation as pseudo probabilities, and identified axles classified by a peak-finding algorithm. Signal section used is the same for each plot with horizontal axis in the samples.

In the present work, Continuous Wavelet Transforms (CWTs) were used because they are generally an effective tool for analysing acoustic and visual signals [23]. In addition, previous work has shown that CWTs are an effective tool for axle identification [18,20,21,22,24].The wavelet transformed signals are subsequently analysed using a Fully Convolutional Network (FCN) that is trained in a supervised manner to perform a binary classification task (Figure 1). As a result, the model outputs a pseudo-probability for each time step, whether a train axle is located above the sensor of the input signal at this time. A peak-finding algorithm is then used to classify the pseudo-probabilities into axle (peak) or no axle (no peak). This enables the processing of signals of arbitrary length without the need to divide them into time windows. Furthermore, analysis in this way is not limited to certain mother wavelets or specific scales, as in the previously mentioned work that used the CWT [18,20,21,22,24].

To validate our method, we recorded a data set on a railway bridge with sensors distributed across the free span of the bridge on the main girders. The impulses of the wheel sets were superimposed with the vibrations of the bridge, which did not allow for their clear visual identification in the time domain. For many bridges and common sensor setups for monitoring purposes, similar to the ones used in this study, the method of Zhu et al. [22] would not be applicable. The VAD, on the other hand, can learn to distinguish the contribution of the structure-dependent natural vibration from the load-induced vibration and can thus be trained for sensors at any point on the bridge. This allows for the application of the method, independent of bridge type and accessibility to specific parts of the bridge.

This paper is structured as follows: In section two, the methods are presented. The first sub-section describes the data acquisition procedure in the field experiment and the subsequent data processing. The second sub-section contains the model definition. In the last sub-section, details on the training of the model are given. Section three presents and discusses the results. The paper ends with section four, in which the conclusions of the present study are drawn.

## 2. Methods

Since we opted to use a supervised learning approach for the VAD, a set of train passages with known axle distances and velocity was required to train the model. In the current research, this information was obtained from strain measurements at the rail level. For future practical applications, the information could be obtained from vehicles with a known axle configuration and through the use of a Differential Global Positioning System (DGPS). If such information is not available, a transfer learning approach based on simulated data could also be an option. In the application, the model can then identify the axles based on the transformed sensor signals. For this purpose, the model first gives a pseudo-probability for the presence of an axle at the longitudinal position of the sensor and for each time step of the analysed signal. In a subsequent step, a peak-finding algorithm is used to extract only the local maxima from the pseudo-probabilities. The extracted maxima represent the time points at which an axle is located above the sensor. As a result, the pseudo-probabilities are classified into axle (class 1) or no axle (class 0), without being limited to classical thresholds (Figure 1). A detailed description is given in the following subsections.

### 2.1. Data Acquisition

**Figure 2:**
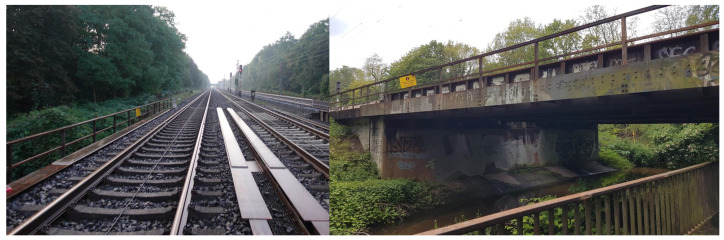
Photos of the investigated structure.

**Figure 3:**
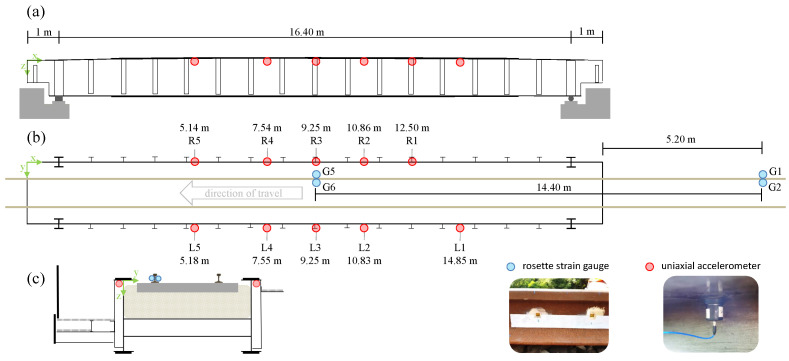
Bridge and sensor setup: (**a**) side view, (**b**) top view with sensor labels, accelerometer *x*-ordinates, and strain gauge distances, (**c**) cross section.

We recorded the measurement data used in the present study on a single-span steel trough railway bridge (Figure 2) located on a long-distance traffic line in Germany. The single-track bridge with a ballasted superstructure was built in 1969 and has a total length of 18.4 m, with a free span of 16.4 m (Figure 3).

The measurement setup is shown in Figure 3. It can be seen that a total of ten seismic uniaxial accelerometers of the type PCB-39B04 (PCB Synotech GmbH, 41836 Hückelhoven, Germany ), with a sensitivity of 1000 mV/g (±10%), a broadband resolution of 0.000003 $g_{\mathrm{RMS}}$, a measurement range of ±5 $g_{\mathrm{pk}}$, and a frequency range of 0.06 to 450 Hz (±5%) were installed. As previously mentioned, we chose an axle detection method based on acceleration measurements because the installation of accelerometers is much easier and less costly compared to strain gauges.

The measurements were triggered from the ring buffer via the rising slope of the wheel load measuring point G1 (Figure 3). The signal was recorded for 60 s, which started ten seconds before the trigger. All sensor signals were recorded with a sampling frequency of $f_{s}=600\;\mathrm{Hz}$ using the catmanAP software and the CX22 data recorder connected to an MX1601B universal amplifier and an MX1616B strain gauge amplifier (all products are from Hottinger Brüel & Kjaer GmbH, 64293 Darmstadt, Germany).

**Figure 4:**
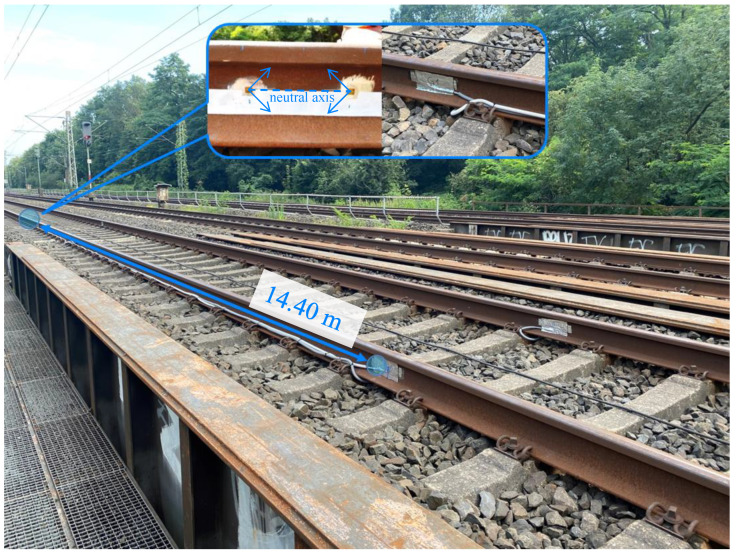
Top view of the bridge with the wheel load measuring points used at a distance of 14.4 m, with a detailed view of the rosette strain gauges and the weatherproof measuring point installed.

By means of two wheel load measuring points, the average velocity of each axle was determined, and from this, the actual position of the axles during the passage was deduced. Every measuring point involved the installation of at least one pair of rosette strain gauges (HBM 1-CXY41-6/350HE) on the rails. Each pair of strain gauges were placed at the level of the neutral axis of the UIC 60 rail profiles with a distance of 20 cm and allowed for the recording of bi-axial strains at an angle of 45° with respect to the neutral axis (Figure 4). Thus, shear strains were obtained. The difference of the shear strains allowed us to determine the acting wheel loads. For further details, please refer to [5]. To compensate for the influence of the lateral wheel loads, a pair of strain gauges was placed on each side of the rail, such that one wheel load measuring point retrieves two signals.

**Figure 5:**
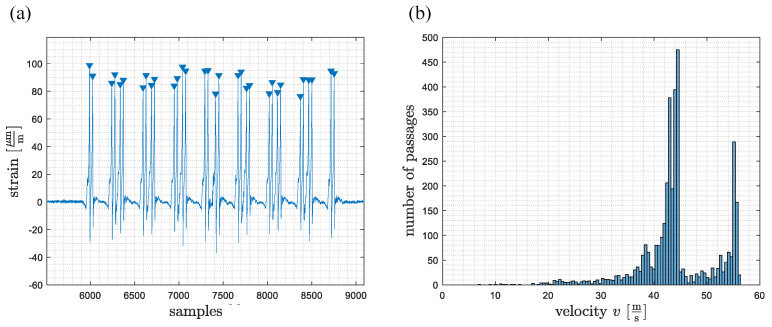
(**a**) Signal of the wheel load measuring point with detected peak values marked with blue triangles (**b**) Histogram of determined mean train velocities for all 3745 passages.

The peaks of the wheel load measurement signals were automatically identified (Figure 5a). All passages where the two wheel load measuring points had not detected the same number of peaks were discarded. This led to 3745 usable recorded passages out of a total of 3787, i.e., about 98.9%. Using the temporal differences of the peaks at the two measuring points and the known distance between the wheel load measurement points of 14.40 m, the mean velocity could be determined for each axle. The trains reached a maximum velocity of about 57 m/s (Figure 5b).

**Figure 6:**
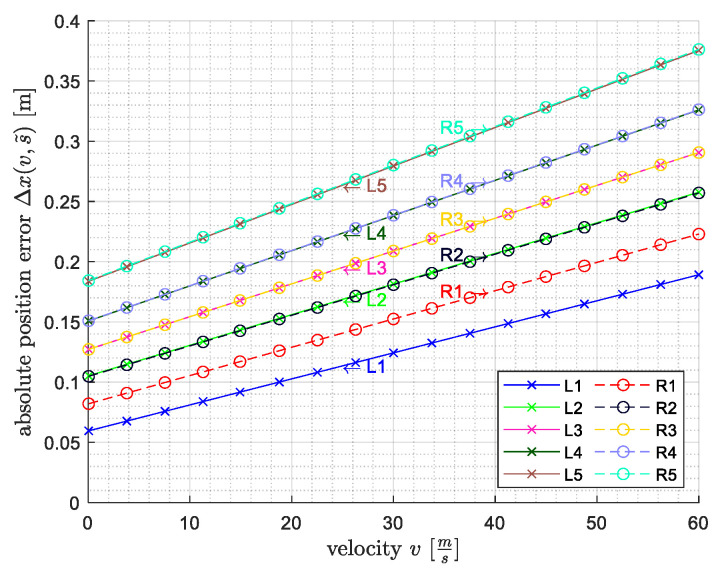
Absolute spatial error of the ground-truth per sensor as a function of velocity.

In the next step, by using the known distances—from the first wheel load measurement point to each of the ten accelerometers—and the mean velocity of each axle, the time at which the axle was at the same *x*-ordinate as the respective sensor could be calculated. Since the two strain gauges of one wheel load measuring point had a distance of 20 cm between them, the uncertainty with respect to the distance between the two wheel load measuring points $s_{\mathrm{WLM}}=14.40\;m$ was assumed to be $\Delta s_{\mathrm{WLM}}=0.2\;m$. This propagated through the velocity determination. Together with an uncertainty in time of $\Delta t=\frac{1}{f_{s}}=\frac{1}{600}\;s$, the absolute spatial error Δx from the linear error propagation for each sensor (Figure 6) was calculated as follows:

(1) $$\Delta x\left(v,\tilde{s}\right)=v\Delta t+\tilde{s}\left(\left|\frac{v}{s_{\mathrm{WLM}}}\right|\Delta t+\left|\frac{1}{s_{\mathrm{WLM}}}\right|\Delta s_{\mathrm{WLM}}\right)$$

This shows that the absolute position error is increased with increasing velocity and with the increasing distance of the sensor with respect to the first wheel load measurement point (G1/G2).

The acceleration signals were combined into one data matrix: $A_{L}^{36,000\times 5}$ for the sensors L1–L5 and $A_{R}^{36,000\times 5}$ for the sensors R1–R5 (Figure 3b), for each passage and without any further signal processing steps. Additionally, two data matrices, $L_{L}^{n_{a}\times 5}$ and $L_{R}^{n_{a}\times 5}$ ($n_{a}$: number of axles), containing the calculated indices at which an axle was at the respective sensor were created.

The complete data set as well as the processing code are available online [25].

### 2.2. Data Transformation

Transforming a signal into the frequency–time domain enables the localisation of frequency content in time [26]. In our case, low-frequency effects such as the bridge’s natural vibration were separated from high-frequency effects such as measurement noise in the frequency domain, while the time domain was preserved. Therefore, the model could learn frequency-specific information, which should lead to faster training and more reliable results.

The most common choices for a frequency–time domain transformation are Short Time Fourier Transformation (STFT) and CWT. The multi-resolution approach of the CWT is particularly useful for complex signals since it adapts the window size to the frequency [27]. The STFT has a fixed resolution, which means that there is always a trade-off between a good time resolution and a good frequency resolution, depending on the window size [26]. As a result, we chose the CWT because it is more suitable for the analysis of acoustic and visual signals than the windowed Fourier transform [23]. The CWT has also been shown in previous work to be an effective tool for axle detection [18,20,21,22,24].

**Figure 7:**
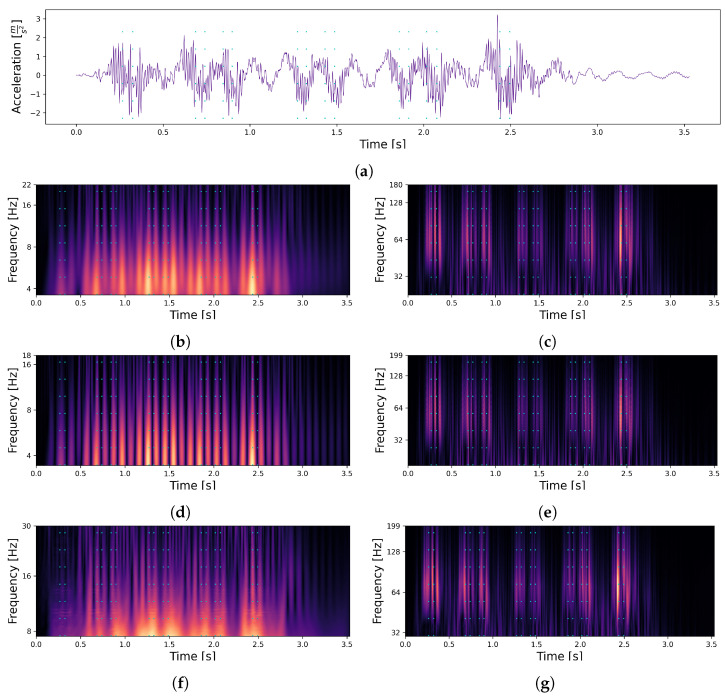
Set of continuous wavelet transformations (CWTs) for the signal obtained from sensor L2 for one of the train passages. The point in time when a load transition occurs is represented by a dashed line in cyan. Each of the transformations were independently normalised from 0 to 1 (visualised with black for 0 and yellow for 1). (**a**) Acceleration signal of a single train passage. (**b**) Complex Gaussian CWT in frequency range of bridge. (**c**) Complex Gaussian CWT in frequency range of axles. (**d**) Gaussian CWT in frequency range of bridge. (**e**) Gaussian CWT in frequency range of axles. (**f**) Frequency B-Spline CWT in frequency range of bridge. (**g**) Frequency B-Spline CWT in frequency range of axles.

With respect to the signals, a section ranging from 150 samples before the first axle to 500 samples after the last axle was further processed and transformed with the PyWavelets package [28] using the determined settings (Table 1). Since the parameter space was too big to be tested on a large scale, the CWT were visualised and analysed for correlations between the axle positions (cyan dotted line) and the power of the transformed signal (Figure 7). As a result, we were able to find that within the range of the bridge’s natural frequency of about 6.9 Hz for the first bending mode (Figure 7 left column), the influence of the bridge on the vibration was mainly visible, while a correlation between the train axles (dashed cyan lines) and the signal did not seem to be present. In the higher frequency range, a correlation became clearer, indicating that the influence of the axles were mainly located in the 64 Hz range (Figure 7 right column).

We assume that it is nevertheless advantageous for the model to receive both pieces of information (influence of the bridge and of the axles) in order to be able to distinguish them better.

As a result, all 6 transformations were used in combination (Figure 7b–g). To create the final model inputs, each signal (per passage and per sensor, shown in Figure 3) was transformed according to our 6 settings. Afterwards, the transformations were independently normalised and stacked into a three-dimensional array $T^{n_{s}\times n_{f}\times n_{t}}$ ($n_{s}$: number of samples, $n_{f}$: number of frequencies/scales, and $n_{t}$: number of transformations). Thus, each sensor functioned independently as a VAD, which means that for our ten sensors, our method can locate each axle ten times (ten time points for ten sensor coordinates).

### 2.3. Model Definition

Our approach for the Virtual Axle Detector (VAD) aimed to always evaluate entire passes in one step so that sufficient context is preserved before and after each axle. Therefore, in our case, the 60 s recordings were always combined into complete passages before evaluation. To ensure that passages of arbitrary length could be efficiently processed, a model with a flexible input length (in the time domain) was essential. Hence, we developed an FCN [29], which only used input size-independent layers such as convolution, pooling, or batch normalization. Our model was developed to output only a single value, between 0 and 1, for the same number of samples as those of the input. These output values represent the model’s certainty for an axle at the *x*-ordinate of the respective sensor.

Our developed VAD model was based on the U-Net architecture, originally proposed by Ronneberger et al. [30], which was developed for semantic segmentation tasks. Here, the goal was to classify each pixel of the input image individually in order to preserve the resolution from the input. For the U-Net, the resolution of the input was halved 4 times (via max pooling) in the encoder path and then doubled 4 times (via transposed convolution) in the decoder path. In addition, the intermediate results before each pooling layer were appended to the intermediate results after the transposed convolution layer with the same resolution, after which they were processed together.

**Figure 8:**
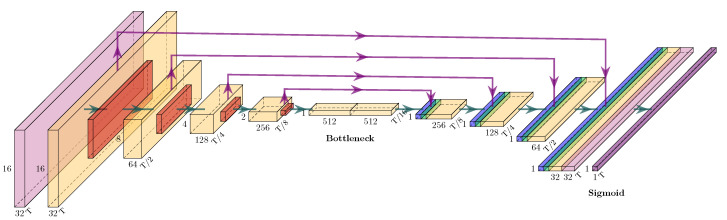
Definition of the Virtual Axle Detection model (VAD), with coloured boxes corresponding to the following layers: CB (light purple), RB (yellow), max pooling (red), concatenate (green), transposed convolution (blue), and reshaping skip connection (purple arrow). Dimensions of the output feature maps for the corresponding layer, with T samples at the bottom right, feature maps at the bottom, and frequencies at the left. The model dimensions for the input: 16 frequencies × 6 transforms × T samples; for the output: 1 × 1 pseudo-probabilities × T samples.

In our case, not each pixel but each sample had to be classified, thus reducing the resolution in the frequency domain to 1. We achieved this by increasing the resolution in the decoder path only in the time dimension (Figure 8), for which we used a transposed convolution layer with a kernel size of 3×1. Before the intermediate results from the encoder path could be appended to the intermediate results from the decoder path, its resolution and number of feature maps were adapted. Each purple arrow in Figure 8 consists of a reshape layer to reduce the frequency domain to the value of 1, and a convolution layer with a kernel size of 1×1 to adapt the number of feature maps.

The convolution blocks (CBs) consist of a batch norm layer and a convolution layer with Rectified Linear Unit (ReLU) activation [31]. The CBs in Figure 8 have a 3×3 kernel size. The residual blocks (RB), originally proposed by He et al. [32], were implemented with 3 CBs in the filtering path and 1 CB in the skip connection. Here, the second CB in the filtering path had a 3×3 kernel size, while the other CBs had a 1×1 kernel size. The results of the filter path and the skip connection were added element-wise before further processing. Our model had 4 pooling steps as the U-Net [30]. We could therefore input transformed signals of any length (in the time domain) as long as they were divisible by 16; the resolution had to remain an integer after being halved 4 times. For lengths that were not multiples of 16, the signal was padded with zeros and thus extended by a maximum of 15 samples.

The last layer is a convolution layer consisting of a single kernel with the size of 3×3 and with sigmoid activation. Therefore, the resulting outputs could be interpreted as independent pseudo probabilities *p*, which indicate the predicted likeliness for a certain class per sample. The resulting model has an input size with an arbitrary number of samples (padded to a multiple of 16), an arbitrary number of signal transformations, and 16 frequencies, which were evenly spaced from the minimum to the maximum scale. The TensorFlow library [33] was used for the implementation of the model, and PlotNeuralNet [34] was used for its visualisation.

### 2.4. Loss Function

We defined the localisation task as a supervised classification problem instead of a regression problem in order to minimise complexity and maximise comprehensibility. We labelled each sample using one of the following classes: Axle at the same *x*-ordinate as the sensor (class 1) or not (class 0).

A common loss function for a binary classification task is Cross Entropy (CE), but for imbalanced data sets, Focal Loss (FL) has been shown to be more effective [35]. In our case, the total number of axles of a train is almost negligible compared to the total amount of samples of a passage. Hence, if the model predicts all values to be 0 (and cannot locate an axle), it will achieve an almost perfect loss for CE and will learn to ignore the axles. This brings us to the thesis that FL is necessary in order to achieve good results. The FL is defined as follows [35]:

(2) $$\mathrm{FL}\left(p_{t}\right)=-1{\left(1-p_{t}\right)}^{\gamma }\log\left(p_{t}\right),$$

where $p_{t}$ is defined as follows:

(3) $$p_{t}=\left\{\begin{array}{cc} p & \mathrm{if}\;y=1\\ 1-p & \mathrm{otherwise}. \end{array}\right.$$

In the equation above, p∈[0,1] is the model’s estimated probability for class 1, *y* is the ground-truth class, and γ is the focusing parameter. The equation of FL consists of $-\log(p_{t})$, which is equal to the CE, and $\left(1-p_{t}\right)$, which is a newly introduced modulating factor weighted by the focusing parameter γ. The larger the factor, the more significant the effect of the modulating factor is, and with a γ of 0, the FL corresponds to the CE [35].

Due to the gamma value, the modulating factor was exponentially included in the equation. As a result, the loss became exponentially smaller, which gave a better prediction. For misclassified examples, the loss was unaffected compared to CE, which made misclassifications much more heavily weighted (a factor of 1000 and higher is possible [35]).

### 2.5. Evaluation Metrics

The loss function itself does not contain information about the number of correctly detected axles. Other metrics are needed to assess the overall performance of the VAD. Accuracy as a metric is also insufficient to draw a conclusion about the model’s performance due to the imbalance in our data set. A prediction containing no axles at all would reach an accuracy of about 99% and would therefore not contain useful information. Precision and recall are suitable metrics for imbalanced data sets [31], but they only take into account binary results and not distance prediction and ground-truth. Due to the high sampling rate and the uncertainty of the labels described in Section 2, however, we wanted to recognise axle predictions within a few samples next to the ground-truth as correct and to measure the temporal error.

**Figure 9:**
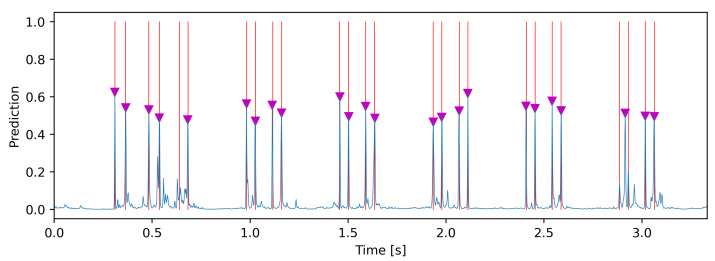
Exemplary output, with the model’s output pseudo-probabilities represented by the blue lines, ground-truth by the red lines, and found peaks by the magenta triangles.

As already mentioned, the model output pseudo probabilities for each time step, whether an axle was on the same *x* coordinate as the respective sensor of the input signal. However, these were continuous values that had to be converted into binary classes (0 for no axle and 1 for with axle). In addition, the model could not natively represent fuzziness and thus often output a large number of small spikes around the ground-truth axles. Thus, in order to obtain a definite point in time for the crossing of an axle over the sensor, despite the uncertainties of the model, the prediction had to be further processed (Figure 9). In order to keep only the meaningful peaks, they were classified using the find-peaks function from SciPy [36]. This function allowed for the use of additional logic for classifications that go beyond setting a threshold. For VAD, we fine-tuned the following parameters of the function: minimum height of the peak (0.25), minimum distance between two peaks (20 samples), and prominence of the peak compared to the surrounding points (0.15). We calculated the minimum distance *d* between two peaks, with an assumed minimum wheel distance $\Delta w_{\min}=2\;m$ and the maximum velocity $v_{\max}=220\;\frac{\mathrm{km}}{h}$, as follows:

(4) $$d=\frac{\Delta w_{\min}\cdot f_{s}}{v_{\max}}\approx \frac{2\;m\cdot 600\;\frac{\mathrm{samples}}{s}}{61.1\;\frac{m}{s}}\approx 20\;\mathrm{samples}$$

A threshold was used to ensure that only predictions within a certain temporal error compared to the ground-truth would be considered correct. For example, the threshold could classify predicted axles as correct with a maximum temporal error of 30 milliseconds compared to the ground-truth. Depending on the application, its requirements may be decisive for the determination of the threshold. In general, it should be taken into account that good results cannot be expected with thresholds that are lower than the label and measurement accuracies. To avoid making assumptions that are too strict, we chose the largest reasonable threshold with 20 samples (Equation (4)) for the first evaluations. After having classified the peaks found as correct or incorrect, they were further evaluated using the following metrics: Precision, recall, and $F_{1}$ score. Precision describes the ratio of true positive predictions to positive predictions and thus allows for a statement regarding how many of the axles have been found. Recall describes the ratio of true positive predictions to false negative predictions and thus allows for a statement in relation to how many of the predicted axles are true. Since both of these metrics only describe a part of the problem, we used the $F_{1}$ score, which is the harmonic mean of precision and recall. Unlike the arithmetic mean, the harmonic mean strongly penalizes small values and thus ensures that axles are neither overlooked nor predicted arbitrarily often.

### 2.6. Optimization of γ

In order to find an optimal γ value for the FL, we performed a parametric study with 150 epochs per run, 150 steps per epoch, and 16 samples per batch. We split the data set randomly, with 70% for training, 20% for validation, and 10% for testing. To ensure comparability, the same random state was used for every run. The selection criterion used for γ was the $F_{1}$ score, because a high $F_{1}$ score indicates a high value for both recall and precision. The complete training logs and graphs for the determination of the γ value are available online [37].

**Figure 10:**
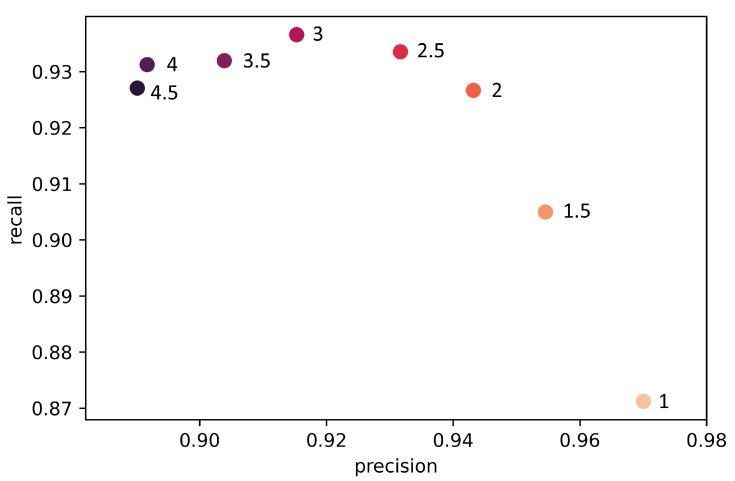
Relationship between γ, precision, and recall with median values of the training results on the validation set.

We thus confirmed our hypothesis that our data set is too unbalanced for standard loss functions such as Cross Entropy. The model training with small γ values of 0 and 0.5 ended in dead ReLUs after 8 or 9 epochs and is therefore unusable. However, the modulation factor should also not be weighted too high to achieve the best performance. The relationship between γ, precision, and recall can be described as a trade-off between detecting too many axles and detecting too few axles (Figure 10).

The γ values of 2, 2.5, and 3 achieved the highest $F_{1}$ score on the validation set. In order to decide which γ value to use for the final evaluation, we trained the model with these γ values in a second run for 300 epochs. In the second run, the γ value of 2.5 achieved the highest $F_{1}$ score (Table 2) and was therefore kept for testing. Since the results of the γ values were close to each other and the middle γ value performed best, we assumed that the optimal value had been found. The complete training logs and graphs for the final models are available online [38].

## 3. Results and Discussion

The test set consisted of 375 train passages with 13,480 axles in total. There were 10 acceleration sensors, for which the individual crossing times had to be determined, resulting in 134,800 times that had to be localised. On the test set, for a threshold of 20 samples, the VAD with a γ value of 2.5 achieved an $F_{1}$ score of 0.938, a recall of 0.946, and a precision of 0.941. Thus, 126,449 out of 134,800 crossing times were localised correctly, with a maximum error of 0.033 s. On average, the predicted axle times had a temporal error of 1.16 samples (0.002 s) compared to the ground-truth, with a standard deviation of 3.06 samples (0.005 s).

Based on the distances between the sensors, we were able to convert the error from samples (temporal) into metres (spatial). In order to examine the spatial error more closely, we chose three threshold values:

- 200 cm as the minimum wheel distance;
- 37 cm as the maximum labelling error (Figure 6);
- 20 cm as the length of the wheel load measuring point.

**Figure 11:**
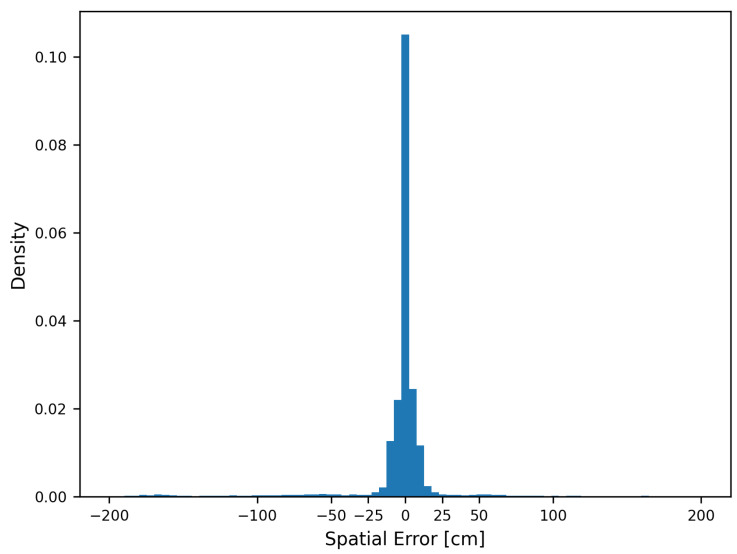
Differences between true and predicted axle positions for a threshold of 2 m.

The spatial errors for a threshold of 2 m were mostly at 0 cm, with an almost symmetrical distribution (Figure 11), thus indicating that there is no bias in the VAD. Most values were within a spatial error of 20 cm, and only a few values had an error higher than 25 cm. The maximum labelling error in the velocity range of most passages (30–60 $\frac{m}{s}$) was partially even above 25 cm (Figure 6).

**Figure 12:**
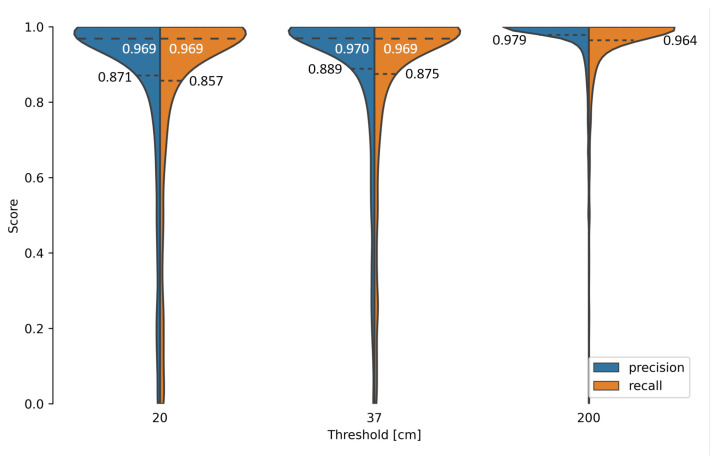
Precision and recall on test data set for different thresholds. Dotted lines with black text represent the 25% quantile, and dashed lines with white text represent the median, if not at 1.0.

We calculated the precision and recall per passage and sensor for each threshold in order to examine the distribution of the metrics in more detail, which resulted in 3750 values per threshold and metric (Figure 12). The differences in the results with thresholds of 20 cm and 37 cm were small, as even the 25% quantile stayed above 85% for both metrics (Figure 12). Precision and recall for a threshold of 200 cm were much better, with the 25% quantile remaining above 96%, while the mean spatial error greatly worsened by more than double the value compared to the other thresholds (Table 3). Therefore, we conclude that 37 cm is the optimal threshold value required to correctly evaluate the model’s performance. In addition, we consider predictions with a spatial error above 37 cm as outliers. It should be possible for such outliers to be sorted out in post-processing by their comparison with known train configurations.

To investigate the influence of sensor placement, we determined the $F_{1}$ score and the spatial accuracy per sensor. The spatial accuracy was calculated as follows:

(5) $$spatial\;accuracy=1-\frac{spatial\;error}{200\;\mathrm{cm}}$$

**Figure 13:**
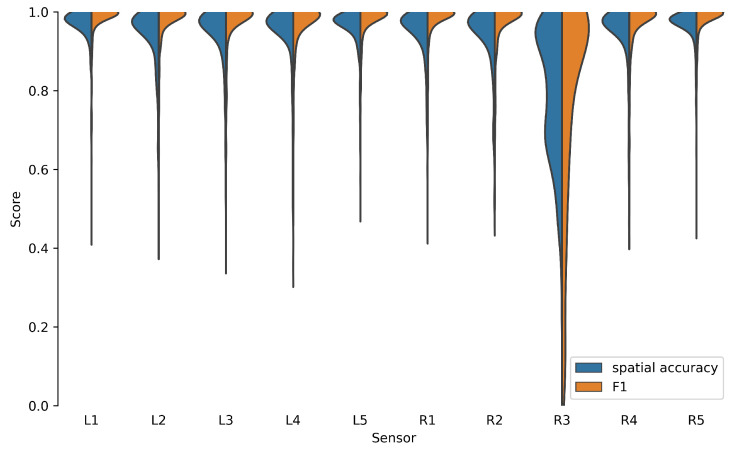
Spatial accuracy (Equation (5)) and $F_{1}$ score on test data set for each sensor (Figure 3).

In this study, it was noticeable that sensor R3 performed significantly worse (Figure 13). In combination with a closer look at the measurement signal of R3, we came to the conclusion that the comparatively poor results were due to a degradation of the sensor. Since the remaining sensors performed comparably well, we concluded that the influence of the sensor placement was negligible.

The evaluation of the test data took 335 s for 375 passages and 10 sensors with the use of an NVIDIA RTX 3090. The model therefore needs 0.089 s per signal, and for our entire measurement setup, 0.89 s per passage. This would allow for the real-time application of the VAD and is a flexible trade-off between accuracy and computing speed due to the number of sensors used.

Compared to the work of Chatterjee et al. [24], who used FAD sensors and wavelets to detect more axles with the FAD, our model shows a comparable success rate in detecting axles. They were able to successfully evaluate 42/47 (about 89.4%) passages. The mean absolute spatial error was about 10.6 cm, which is about three times as much as that in our study. The achieved spatial accuracy in our study is still 1.4 times better compared to that obtained in a study using FAD sensors combined with an optimized mother wavelet and wavelet scale for the identification of axles [20]. Taking into account that we did not use FAD in our method, and that the velocities were about twice as high, this is a confirmation of our hypothesis that it is advantageous not to limit the analysis to certain mother wavelets and certain scales. In contrast to the method of Zhu et al. [22], due to our model architecture, the VAD can be applied at any point of the bridge. This allows for the use of common SHM measurement setups in axle detection without the need to attach additional sensors. The accuracies of the methods are similar. It should be noted that in all cases, the detection of car axles is compared with that of train axles.

## 4. Conclusions

We demonstrated that with our proposed method, no additional FADs or strain gauges on the main girders are required to realise a NOR-BWIM system. Instead, our method allows for accelerometers at any point of the structure to be used as VADs because our model can learn to account for the influence of the bridge structure and the sensor placement. As a result, the much more complex installation of strain gauges as well as track closures can be avoided.

We were also able to show that FCNs can detect axles using only acceleration measurements within a spatial accuracy of 37 cm, with a precision of 93% and a recall of 91%. The mean value of the absolute values of the spatial errors compared to the ground-truth here is about 3.9 cm. The results showed that the method can detect axles with spatial errors similar to the data used for labelling.

Even though our results show higher accuracy compared to other studies that used different methodologies, we assume that the accuracy of determining the vehicle configuration and velocity could be increased through the joint evaluation of several sensors, increased model complexity, improved signal transformation, or the use of different measured quantities such as strain and displacement. Enabling the method to be used with other measured quantities would also increase the amount of use cases.

Finally, the most important issue is the generalisability of the model. How efficiently the model can be used depends on whether it needs to be re-trained to be able to use the method, and if so, whether real or simulated data should be employed. Should retraining with real data be necessary, we propose to determine the axle position during the passages using vehicles with known axle configuration and DGPS.

## Figures and Tables

**Table 1:** Continuous wavelet transformation settings.

Wavelet	Figure	Lower Scale Limit	Upper Scale Limit
First Order Complex Gaussian Derivative	Figure 7b	1	8
	Figure 7c	8	50
First Order Gaussian Derivative	Figure 7d	0.6	6.5
	Figure 7e	6.5	35
Default Frequency B-Spline [28]	Figure 7f	1.5	10
	Figure 7g	10	40

**Table 2:** The model’s performance on the validation set, depending on the γ value of FL with increased training length. Each of the precision and recall values was taken from the epoch with the highest $F_{1}$ value.

γ	$F_{1}$	Precision	Recall
3	0.9538	0.9477	0.9620
2.5	0.9544	0.9556	0.9542
2	0.9534	0.9559	0.9522

**Table 3:** Influence of the threshold on mean spatial error, $F_{1}$, precision, and recall.

Threshold (cm)	Mean (cm)	$F_{1}$	Precision	Recall
200	10.3	0.954	0.970	0.948
37	3.9	0.915	0.926	0.910
20	3.5	0.897	0.905	0.892

## Data Availability

The data [25] as well as the source code [39] used in this paper are published and contain: (1) All measurement data; (2) Matlab code to label data and save as text files; (3) Python code for transformation, training, evaluation, and plotting. In addition, the training logs are also available for the determination of the gamma value [37] and for the final training [38].

## Abbreviations

The following abbreviations were used in this manuscript:

BWIM	Bridge Weigh-In-Motion
CB	Convolution block
CE	Cross Entropy
CNN	Convolutional Neural Network
CWT	Continuous-Wavelet-Transformation
DGPS	Differential Global Positioning System
FAD	Free-of-axle-detector
FCN	Fully Convolutional Network
FL	Focal Loss
NOR	Nothing-on-road
RB	Residual block
ReLU	Rectified Linear Unit
SHM	Structural health monitoring
STFT	Short Time Fourier Transformation
VAD	Virtual Axle Detector

## References

[1] ASCE (2021). Structurally Deficient Bridges | Bridge Infrastructure | ASCE’s 2021 Infrastructure Report Card. https://infrastructurereportcard.org/cat-item/bridges-infrastructure/.

[2] Geißler K. (2014). Front Matter.

[3] Knapp N. (2019). Brücken bei der Deutschen Bahn. https://www.deutschebahn.com/de/presse/suche_Medienpakete/medienpaket_bruecken-6854340.

[4] Chan T., Yu L., Law S., Yung T. (2001). Moving Force Identification Studies, I: Theory. J. Sound Vib..

[5] Kouroussis G., Caucheteur C., Kinet D., Alexandrou G., Verlinden O., Moeyaert V. (2015). Review of Trackside Monitoring Solutions: From Strain Gages to Optical Fibre Sensors. Sensors.

[6] Firus A., Kemmler R., Berthold H., Lorenzen S., Schneider J. (2022). A time domain method for reconstruction of pedestrian induced loads on vibrating structures. Mech. Syst. Signal Process..

[7] Kazemi Amiri A., Bucher C. (2017). A procedure for in situ wind load reconstruction from structural response only based on field testing data. J. Wind. Eng. Ind. Aerodyn..

[8] Hwang J., Kareem A., Kim W. (2009). Estimation of modal loads using structural response. J. Sound Vib..

[9] Lourens E., Papadimitriou C., Gillijns S., Reynders E., De Roeck G., Lombaert G. (2012). Joint input-response estimation for structural systems based on reduced-order models and vibration data from a limited number of sensors. Mech. Syst. Signal Process..

[10] Firus A. (2022). A Contribution to Moving Force Identification in Bridge Dynamics. Ph.D. Thesis.

[11] Lydon M., Robinson D., Taylor S.E., Amato G., Brien E.J.O., Uddin N. (2017). Improved axle detection for bridge weigh-in-motion systems using fiber optic sensors. J. Civ. Struct. Health Monit..

[12] Wang H., Zhu Q., Li J., Mao J., Hu S., Zhao X. (2019). Identification of moving train loads on railway bridge based on strain monitoring. Smart Struct. Syst..

[13] Yu Y., Cai C., Deng L. (2016). State-of-the-art review on bridge weigh-in-motion technology. Adv. Struct. Eng..

[14] He W., Ling T., OBrien E.J., Deng L. (2019). Virtual Axle Method for Bridge Weigh-in-Motion Systems Requiring No Axle Detector. J. Bridge Eng..

[15] Thater G., Chang P., Schelling D.R., Fu C.C. (1998). Estimation of bridge static response and vehicle weights by frequency response analysis. Can. J. Civ. Eng..

[16] Zakharenko M., Frøseth G.T., Rönnquist A. (2022). Train Classification Using a Weigh-in-Motion System and Associated Algorithms to Determine Fatigue Loads. Sensors.

[17] Bernas M., Płaczek B., Korski W., Loska P., Smyła J., Szymała P. (2018). A Survey and Comparison of Low-Cost Sensing Technologies for Road Traffic Monitoring. Sensors.

[18] Yu Y., Cai C., Deng L. (2017). Vehicle axle identification using wavelet analysis of bridge global responses. J. Vib. Control..

[19] O’Brien E.J., Hajializadeh D., Uddin N., Robinson D., Opitz R. Strategies for Axle Detection in Bridge Weigh-in-Motion Systems. Proceedings of the International Conference on Weigh-In-Motion.

[20] Zhao H., Tan C., OBrien E.J., Uddin N., Zhang B. (2020). Wavelet-Based Optimum Identification of Vehicle Axles Using Bridge Measurements. Appl. Sci..

[21] Kalhori H., Alamdari M.M., Zhu X., Samali B., Mustapha S. (2017). Non-intrusive schemes for speed and axle identification in bridge-weigh-in-motion systems. Meas. Sci. Technol..

[22] Zhu Y., Sekiya H., Okatani T., Yoshida I., Hirano S. (2021). Acceleration-Based Deep Learning Method for Vehicle Monitoring. IEEE Sensors J..

[23] Daubechies I. (1990). The wavelet transform, time-frequency localization and signal analysis. IEEE Trans. Inf. Theory.

[24] Chatterjee P., OBrien E., Li Y., González A. (2006). Wavelet domain analysis for identification of vehicle axles from bridge measurements. Comput. Struct..

[25] Lorenzen S.R., Riedel H., Rupp M., Schmeiser L., Berthold H., Firus A., Schneider J. (2022). Virtual Axle Detector based on Analysis of Bridge Acceleration Measurements by Fully Convolutional Network. arXiv.

[26] Brunton S.L., Kutz J.N. (2019). Data-Driven Science and Engineering: Machine Learning, Dynamical Systems, and Control.

[27] Mallat S. (1989). A theory for multiresolution signal decomposition: The wavelet representation. IEEE Trans. Pattern Anal. Mach. Intell..

[28] Lee G.R., Gommers R., Waselewski F., Wohlfahrt K., O’Leary A. (2019). PyWavelets: A Python package for wavelet analysis. J. Open Source Softw..

[29] Long J., Shelhamer E., Darrell T. Fully convolutional networks for semantic segmentation. Proceedings of the 2015 IEEE Conference on Computer Vision and Pattern Recognition (CVPR).

[30] Ronneberger O., Fischer P., Brox T. (2015). U-Net: Convolutional Networks for Biomedical Image Segmentation. Proceedings of the Medical Image Computing and Computer-Assisted Intervention—MICCAI 2015.

[31] Géron A. (2019). Hands-On Machine Learning with Scikit-Learn, Keras, and TensorFlow: Concepts, Tools, and Techniques to Build Intelligent Systems.

[32] He K., Zhang X., Ren S., Sun J. Deep Residual Learning for Image Recognition. Proceedings of the 2016 IEEE Conference on Computer Vision and Pattern Recognition (CVPR).

[33] Abadi M., Agarwal A., Barham P., Brevdo E., Chen Z., Citro C., Corrado G.S., Davis A., Dean J., Devin M. (2015). TensorFlow: Large-Scale Machine Learning on Heterogeneous Systems. https://www.tensorflow.org/.

[34] Iqbal H. (2018). HarisIqbal88/PlotNeuralNet v1.0.0.

[35] Lin T.Y., Goyal P., Girshick R., He K., Dollár P. Focal Loss for Dense Object Detection. Proceedings of the 2017 IEEE International Conference on Computer Vision (ICCV).

[36] Virtanen P., Gommers R., Oliphant T.E., Haberland M., Reddy T., Cournapeau D., Burovski E., Peterson P., Weckesser W., Bright J. (2020). SciPy 1.0: Fundamental Algorithms for Scientific Computing in Python. Nat. Methods.

[37] Riedel H. (2022). Training Logs for Determination of the Gamma Value. https://www.comet.com/imsdcomet/vader.

[38] Riedel H. (2022). Training Logs for the Final Models. https://www.comet.com/imsdcomet/vader2.

[39] Riedel H., Rupp M. (2022). VADer.

